# Supplementary material for: Structure and function of the mycobacterial transcription initiation complex with the essential regulator RbpA
Source: eLife. 2017 Jan 9;6:e22520. doi: 10.7554/eLife.22520 (PMC5302886; doi:10.7554/eLife.22520)
Supplement: Supplementary file 1. — DOI: http://dx.doi.org/10.7554/eLife.22520.012 [file elife-22520-supp1.docx]

**Supplementary file 1. Sequence identity of Msm, Mtb, and Mbo transcription initiation proteins.**

| subunit | Total residues (*Msm*) | Sequence identity (%; *Msm* vs *Mtb*) | Sequence identity (%; *Mbo* vs *Mtb*) |
| --- | --- | --- | --- |
| α | 700 | 91.6 | 100 |
| β | 1,169 | 91.2 | 99.9 |
| β’ | 1,317 | 91.6 | 99.9 |
| ω | 107 | 81.3 | 100 |
| Core RNAP | 3,293 | 91.1 | 99.9 |
| σ^A^ | 466 | 86.1 | 100 |
| RNAP holoenzyme | 3,759 | 90.5 | 99.9 |
| CarD | 162 | 98.1 | 100 |
| RbpA | 114 | 91.9 | 100 |
| Transcription factors | 276 | 95.5 | 100 |
| total | 4,035 | 90.8 | 99.9 |
