# Supplementary material for: Structure and function of the mycobacterial transcription initiation complex with the essential regulator RbpA
Source: eLife. 2017 Jan 9;6:e22520. doi: 10.7554/eLife.22520 (PMC5302886; doi:10.7554/eLife.22520)
Supplement: Supplementary file 2. — DOI: http://dx.doi.org/10.7554/eLife.22520.013 [file elife-22520-supp2.docx]

**Supplementary file 2. Table of crystallographic statistics.**

|  | *Msm* RbpA/TIC |
| --- | --- |
| **Data collection** |  |
| Space group | P2_1_ |
| Combined datasets | 4 |
| Cell dimensions |  |
| *a* (Å) | 133.012 |
| *b* (Å) | 161.633 |
| *c* (Å) | 139.211 |
|  |  |
| Wavelength (Å) | 0.97918 |
| Resolution (Å) | 51.99 – 2.76 (2.859 – 2.76)^a^ |
| Total reflections | 2,329,541 (175,429) |
| Unique reflections | 143,776 (13,955) |
| Multiplicity | 16.2 (12.3) |
| Completeness (%) | 99 (100) |
| <*I*>/σ*I* | 22.39 (0.77) |
| Wilson B-factor (Å^2^) | 78.20 |
| *R*_merge_^b^ | 0.2343 (4.816) |
| *R*_meas_^b^ | 0.2417 (5.021) |
| *R*_pim_^b^ | 0.059 (1.564) |
|  |  |
| CC1/2^c^ | 0.998 (0.214) |
| CC*^c^ | 1 (0.594) |
|  |  |
| **Refinement** |  |
| *R*_work_ / *R*_free_ | 0.2388/0.2795 (0.4515/0.4630) |
| CC_work_/CC_free_^c^ | 0.952/0.929 (0.467/0.437) |
| No. atoms | 26,608 |
| Macromolecule | 26,396 |
| Ligand/ion | 87 |
| Water | 125 |
| Protein residues | 3,329 |
| *B*-factors |  |
| Macromolecules | 85.92 |
| Ligand/ions/water | 80.82 |
| R.m.s deviations |  |
| Bond lengths (Å) | 0.004 |
| Bond angles (°) | 0.74 |
| Clashscore | 36.27 |
| Ramachandran favored (%) | 96 |
| Ramachandran outliers (%) | 0.27 |

^a^ Values in parentheses are for highest-resolution shell.

**^b^** (Diederichs and Karplus, 1997)

^c^ (Karplus and Diederichs, 2012)
