# Supplementary material for: Structure and function of the mycobacterial transcription initiation complex with the essential regulator RbpA
Source: eLife. 2017 Jan 9;6:e22520. doi: 10.7554/eLife.22520 (PMC5302886; doi:10.7554/eLife.22520)
Supplement: Supplementary file 3. — DOI: http://dx.doi.org/10.7554/eLife.22520.014 [file elife-22520-supp3.docx]

**Supplementary file 3. Conditions for kinetic experiments with Cy3-AP3.**

| Sample | [Cy3-AP3]  (nM) | [RNAP]  (nM) | [RbpA]  (nM) | [CarD]  (nM) |
| --- | --- | --- | --- | --- |
| *Eco* holo | 1 | 5, 10, 25, 50, 100, 200, 400 |  |  |
| *Mbo* holo | 1 | 3, 5, 10, 25, 50, 100, 200, 400 |  |  |
| *Mbo* holo+RbpA | 1 | 3, 5, 10, 25, 50, 100, 200, 400 | 5,000 |  |
| *Mbo* holo+CarD | 1 | 3, 5, 10, 25, 50, 100, 200, 400 |  | 10,000 |
| *Mbo* holo+RbpA+CarD | 1 | 3, 5, 10, 25, 50, 100, 200 | 5,000 | 10,000 |
