# Supplementary material for: Structure and function of the mycobacterial transcription initiation complex with the essential regulator RbpA
Source: eLife. 2017 Jan 9;6:e22520. doi: 10.7554/eLife.22520 (PMC5302886; doi:10.7554/eLife.22520)
Supplement: Supplementary file 4. — DOI: http://dx.doi.org/10.7554/eLife.22520.015 [file elife-22520-supp4.docx]

**Supplementary file 4. Unconstrained kinetic parameters on the *Mtb* AP3 promoter.**


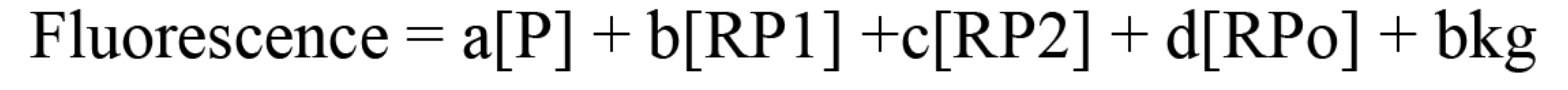
The fluorescence progress curves (Figures 3A-C, S3B-C) were fit according to the 3-step sequential kinetic scheme:

$$R +P \begin{matrix} k_{1} \\ \rightleftarrows\\ k_{-1} \end{matrix} RP1 \begin{matrix} k_{2} \\ \rightleftarrows\\ k_{-2} \end{matrix} RP2 \begin{matrix} k_{3} \\ \rightleftarrows\\ k_{-3} \end{matrix}\mathrm{RPo}$$

The following equation was used to describe the fluorescence signal (the observable):

$$Fluorescence=a\left[ P \right]+ b\left[ RP1 \right]+ c\left[ RP2 \right]+ d\left[ \mathrm{RPo} \right]+ \mathrm{bkg}$$

P (free Cy3-AP3 promoter DNA; Figure S3A), and the DNA-bound RNAP species RP1, RP2, and RPo are the fluorescent species present in the kinetic scheme. The fluorescence scale factors a, b, c, and d account for different fluorescence yields for each species (because of the changing Cy3 environment).

|  | RNAP | | | | |  |
| --- | --- | --- | --- | --- | --- | --- |
|  | *Mbo* holo | | | | *Eco* holo | Std. dev. (% of mean value)^g^ |
| parameter |  | +RbpA | + CarD | +CarD+RbpA |  |  |
| n^a^ | 3^b^ | 3^b^ | 2^b^ | 2^b^ | 1^c^ |  |
| *k*_1_ (M^-1^s^-1^) | (9.8 ± 0.6) x 10^6^ | (1.7 ± 0.3) x 10^7^ | (5.3 ± 0.5) x 10^7^ | (1.2 ± 0.1) x 10^8^ | (1.2 ± 0.1) x 10^8^ | 84 |
| *k*_-1_ (s^-1^) | 2.6 ± 0.2 | 1.6 ± 0.4 | 5.3 ± 0.9 | 33 ± 13 | 3.4 ± 0.5 | 150 |
| K_1_ (M^-1^)^d^ | (3.7 ± 0.5) x 10^6^ | (1.0 ± 0.3) x 10^7^ | (1.0 ± 0.2) x 10^7^ | > (3.5 ± 1.4) x 10^6^ | > (3.5 ± 0.6) x 10^7^ |  |
| *k*_2_ (s^-1^) | 0.40 ± 0.02 | 1.5 ± 0.2 | 3.6 ± 0.5 | 10 ± 0.7 | 1.2 ± 0.03 | 120 |
| *k*_-2_ (s^-1^) | 0.047 ± 0.005 | 0.095 ± 0.019 | 0.062 ± 0.005 | 0.033 ± 0.002 | 0.11 ± 0.01 | 47 |
| K_2_ | 8.5 ± 1.0 | 15 ± 4 | 58 ± 9 | 310 ± 27 | 11 ± 1 |  |
| *k*_3_ (s^-1^) | 0.049 ± 0.007 | 0.063 ± 0.011 | 0.064 ± 0.011 | 0.10 ± 0.03 | 0.084 ± 0.015 | 26 |
| *k*_-3_ (s^-1^) | 0.013 ± 0.003 | 0.013 ± 0.002 | 2.0 x 10^-3^ | (1.2 ± 0.2) x 10^-3^ | 8.9 x 10^-7^ | 110 |
| K_3_ | 3.8 ± 1.0 | 4.9 ± 1.2 | 32 ± 5 | 89 ± 27 | (9.4 ± 1.7) x 10^4^ |  |
| K_1_K_2_K_3_ (M^-1^) | (1.2 ± 0.5) x 10^8^ | (7.8 ± 3.5) x 10^8^ | (1.8 ± 0.6) x 10^10^ | (9.8 ± 4.9) x 10^10^ | 3.6 x 10^13^ |  |
| *k*_d_ (s^-1^)^e^ | (5.6 ± 0.8) x 10^-3^ | (6.9 ± 3.1) x 10^-3^ | (9.5 ± 5.9) x 10^-4^ | (2.8 ± 0.8) x 10^-4^ | 5.0 x 10^-7^ |  |
| t_1/2_ (min)^e^ | 2.1 ± 0.3 | 1.7 ± 0.8 | 12 ± 7 | 42 ± 13 | 2.3 x 10^4^ |  |
| t_1/2_^exp^ (min)^f^ | ~2 | ~1.5 | ~10 | ~30 | >> 60 |  |
| a | 0.30 ± 0.02 | 0.20 ± 0.02 | 0.27 ± 0.01 | 0.32 ± 0.04 | 0.28 | 17 |
| b | 0.50 ± 0.02 | 0.61 ± 0.04 | 0.49 ± 0.04 | 0.26 ± 0.02 | 0.39 | 29 |
| c | 1.2 ± 0.1 | 1.2 ± 0.1 | 1.3 ± 0.1 | 1.3 ± 0.1 | 1.1 | 7 |
| d | 1.2 ± 0.1 | 1.2 ± 0.1 | 1.3 ± 0.1 | 1.3 | 1.2 | 4 |

Color coding:

Light green: 5 – 10-fold > *Mbo* holo Light red: 5 – 10-fold < *Mbo* holo

Dark green: > 10-fold over *Mbo* holo Dark red: more than 10-fold < *Mbo* holo

^a^ Number of independent trials.

^b^ The values listed for *k*_1_, *k*_-1_, *k*_2_, *k*_-2_, *k*_3_, *k*_-3_, a, b, c, and d (parameters determined directly from fitting the raw fluorescence progress curves) are the means across the independent trials. The errors listed are the standard errors across the independent trials.

^c^ For *Eco* holo, n = 1. The values listed for *k*_1_, *k*_-1_, *k*_2_, *k*_-2_, *k*_3_, *k*_-3_, a, b, c, and d were determined directly from fitting the raw fluorescence progress curves (Figure 3A). The errors listed are the standard errors from the global fit as reported by Kintek Global Kinetic Explorer (Johnson et al., 2009a).

^d^ The values for K_1_, K_2_, K_3_ were calculated from the fitted parameters: K_1_ = *k*_1_/*k*_-1_, K_2_ = *k*_2_/*k*_-2_, K_3_=*k*_3_/*k*_-3_. The errors were calculated by propagation of errors from the errors in the fitted parameters.

^e^ The value for *k*_d_, the dissociation rate for RPo, was calculated using equation (17) of (Tsodikov and Record, 1999):

$$\frac{1}{k_{d}}= \frac{1}{k_{-3}}+ \frac{1+ K_{3}}{k_{-2}}+ \frac{K_{2}+ K_{2}K_{3}}{k_{-1}}+ \frac{1}{k_{-1}} (2)$$

The errors were calculated by propagation of errors. The value for t_1/2_ was calculated as t_1/2_ = ln(2)/*k*_d_ (errors calculated by propagation of errors).

^f^ The experimental half-life (t_1/2_^exp^) was determined from promoter lifetime experiments (Figure 2C).

^g^ The standard deviation (σ_x_) as a % of the mean value of a fitted parameter (<x>) was calculated as follows:

% std. dev. = 100(σ_x_/<x>)
