# Supplementary material for: Structure and function of the mycobacterial transcription initiation complex with the essential regulator RbpA
Source: eLife. 2017 Jan 9;6:e22520. doi: 10.7554/eLife.22520 (PMC5302886; doi:10.7554/eLife.22520)
Supplement: Supplementary file 5. — DOI: http://dx.doi.org/10.7554/eLife.22520.016 [file elife-22520-supp5.docx]

**Supplementary file 5. Conditions for kinetic experiments with Cy3-VapB.**

| Sample | [Cy3-VapB]  (nM) | [RNAP]  (nM) | [RbpA]  (nM) |
| --- | --- | --- | --- |
| *Eco* holo | 2 | 10, 25, 50, 250, 500 |  |
| *Mbo* holo | 2 | 6, 10, 25, 50, 100, 250, 400, 500 |  |
| *Mbo* holo+RbpA | 2 | 6, 10, 25, 50, 100, 250, 400, 500 | 5,000 |
| *Mbo* holo+RbpA^CD-BL-SID^ | 2 | 10, 25, 50, 100, 250, 500 | 5,000 |
| *Mbo* holo+RbpA^BL-SID^ | 2 | 6, 10, 25, 50, 100, 250, 400, 500 | 5,000 |
| *Mbo* holo+RbpA^R79A^ | 2 | 6, 10, 25, 50, 100, 250, 400, 500 | 5,000 |
