# Supplementary material for: Structure and function of the mycobacterial transcription initiation complex with the essential regulator RbpA
Source: eLife. 2017 Jan 9;6:e22520. doi: 10.7554/eLife.22520 (PMC5302886; doi:10.7554/eLife.22520)
Supplement: Supplementary file 6. — VapB promoters. DOI: http://dx.doi.org/10.7554/eLife.22520.017 [file elife-22520-supp6.docx]

**Supplementary file 6. Comparison of kinetic parameters for AP3 vs. VapB promoters.**

| RNAP | *Mbo* | | | | ** | | |
| --- | --- | --- | --- | --- | --- | --- | --- |
|  |  |  | +RbpA | +RbpA |  |  |  |
| promoter | AP3 | VapB | AP3 | VapB | AP3 | VapB |  |
| *k*_1_ (M^-1^s^-1^) | 1.1 x 10^7^ | 1.9 x 10^7^ | 1.7 x 10^7^ | 1.5 x 10^7^ | 1.2 x 10^8^ | 2.8 x 10^7^ |  |
| *k*_-1_ (s^-1^) | 2.1 | 1.4 | 1.3 | 2.3 | 3.4 | 1.8 |  |
| K_1_ (M^-1^) | 5.2 x 10^6^ | 1.4 x 10^7^ | 1.3 x 10^7^ | 6.5 x 10^6^ | > 3.5 x 10^7^ | 1.6 x 10^7^ |  |
| *k*_2_ (s^-1^) | 0.36 | 7.7 x 10^-3^ | 1.3 | 0.18 | 1.2 | 5.3 x 10^-3^ |  |
| *k*_-2_ (s^-1^) | 0.041 | 6.1 x 10^-3^ | 0.13 | 0.034 | 0.11 | 3.4 x 10^-3^ |  |
| K_2_ | 8.8 | 1.3 | 10 | 5.3 | 11 | 1.6 |  |
| *k*_3_ (s^-1^) | 0.035 | 0.035 | 0.082 | 0.010 | 0.083 | 0.026 |  |
| *k*_-3_ (s^-1^) | 0.014 | 9.8 x 10^-3^ | 0.013 | 2.3 x 10^-3^ | 0 | 4.9 x 10^-3^ |  |
| K_3_ | 2.5 | 3.6 | 6.3 | 4.3 | - | 5.3 |  |
| K_1_K_2_K_3_ | 1.2 x 10^8^ | 6.1 x 10^7^ | 8.3 x 10^8^ | 1.5 x 10^8^ | - | 1.3 x 10^8^ |  |
| *k*_d_^a^ | 6.3 x 10^-3^ | 1.1 x 10^-3^ | 7.1 x 10^-3^ | 1.7 x 10^-3^ | - | 4.8 x 10^-4^ |  |
| t_1/2_ (min) | 1.8 | 9.8 | 1.6 | 6.8 | - | 24 |  |
| t_1/2_^exp^ (min) | ~2 | ~5 | ~1.5 | ~4 | >> 60 |  |  |
