# Supplementary material for: Structure and function of the mycobacterial transcription initiation complex with the essential regulator RbpA
Source: eLife. 2017 Jan 9;6:e22520. doi: 10.7554/eLife.22520 (PMC5302886; doi:10.7554/eLife.22520)
Supplement: Supplementary file 8. — DOI: http://dx.doi.org/10.7554/eLife.22520.019 [file elife-22520-supp8.docx]

**Supplementary file 8. Kinetic parameters on the *Mtb* VapB promoter.**

| RNAP | *Mbo* holo | | | | | *Eco* holo |
| --- | --- | --- | --- | --- | --- | --- |
|  |  | +RbpA | +RbpA^CD-BL-SID^ | +RbpA^BL-SID^ | +RbpA^R79A^ |  |
| *k*_1_ (M^-1^s^-1^) | 1.9 x 10^7^ | 1.5 x 10^7^ | 1.2 x 10^7^ | 1.8 x 10^7^ | 1.2 x 10^7^ | 2.8 x 10^7^ |
| *k*_-1_ (s^-1^) | 1.4 | 2.3 | 3.6 | 3.5 | 0.65 | 1.8 |
| K_1_ (M^-1^) | 1.4 x 10^7^ | 6.5 x 10^6^ | 3.3 x 10^6^ | 5.1 x 10^6^ | 1.9 x 10^7^ | 1.6 x 10^7^ |
| *k*_2_ (s^-1^) | 7.7 x 10^-3^ | 0.18 | 0.20 | 0.40 | 5.0 x 10^-3^ | 5.3 x 10^-3^ |
| *k*_-2_ (s^-1^) | 6.1 x 10^-3^ | 0.034 | 0.033 | 0.044 | 0.010 | 3.4 x 10^-3^ |
| K_2_ | 1.3 | 5.3 | 6.1 | 9.1 | 0.50 | 1.6 |
| *k*_3_ (s^-1^) | 0.035 | 0.010 | 0.017 | 0.016 | 0.022 | 0.026 |
| *k*_-3_ (s^-1^) | 9.8 x 10^-3^ | 2.3 x 10^-3^ | 3.1 x 10^-3^ | 4.5 x 10^-3^ | 9.9 x 10^-3^ | 4.9 x 10^-3^ |
| K_3_ | 3.6 | 4.3 | 5.5 | 3.6 | 2.2 | 5.3 |
| K_1_K_2_K_3_ | 6.1 x 10^7^ | 1.5 x 10^8^ | 1.1 x 10^8^ | 1.7 x 10^8^ | 2.1 x 10^7^ | 1.3 x 10^8^ |
| *k*_d_^a^ | 1.1 x 10^-3^ | 1.7 x 10^-3^ | 1.9 x 10^-3^ | 3.1 x 10^-3^ | 2.3 x 10^-3^ | 4.8 x 10^-4^ |
| t_1/2_ (min) | 9.8 | 6.8 | 6.0 | 3.8 | 4.9 | 24 |
