# Supplementary material for: Structure and function of the mycobacterial transcription initiation complex with the essential regulator RbpA
Source: eLife. 2017 Jan 9;6:e22520. doi: 10.7554/eLife.22520 (PMC5302886; doi:10.7554/eLife.22520)
Supplement: Supplementary file 9. — (A) Table of Msm strains used in this study. (B) Table of oligos used in generating Msm strains. DOI: http://dx.doi.org/10.7554/eLife.22520.020 [file elife-22520-supp9.docx]

**Supplementary file 9A *Msm* strains used in this study.**

| Strain name | genotype | construction |
| --- | --- | --- |
| MGM6029 | *rpoC:rpoC-ppx-10his hyg* | MC^2^155 transformed with pAJF527 |
| MGM6221 | *rbpA::rbpA hyg galK sacB (rbpA KO intermediate)* | MC2155 transformed with pAJF674 |
| MGM6223 | *attB:rbpA strep rbpA::rbpA hyg galK sacB (rbpA KO intermediate)* | MGM6221 transformed with pAJF685 |
| MGM6228 | *ΔrbpA attB::rbpA strep* | MGM6223 passaged in sucrose/2DOG media for negative selection of integration |
| MGM6232 | *ΔrbpA attB::rbpA kan* | MGM6228 transformed with pAJF672 |
| MGM6234 | *ΔrbpA attB::rbpA(28-114) kan* | MGM6228 transformed with pAJF679 |
| MGM6236 | *ΔrbpA attB::rbpA(72-114) kan* | MGM6228 transformed with pAJF680 |
| MGM6273 | *ΔrbpA attB::rbpA(R79A) kan* | MGM6228 transformed with pAJF736 |

**Supplementary file 9B. Oligos used in generating *Msm* strains.**

| Name | 5'-3' sequence |
| --- | --- |
| 306knfwd | ctgtggataaccgtattaccgcc |
| oAF020 | TAATACGACTCACTATAGGG (T7 universal) |
| oAF021 | CAATTAACCCTCACTAAAGG (T3 universal) |
| oAF765 | gtcgttcacggctctagc |
| oAF796 | GTGATGGCCGCTGCTGGGCCCCTGGAACAGAACTTCCAGgcggtaatccgagtag |
| oAF797 | CCCAGCAGCGGCCATCACCATCACCATCATCACCATCACCATTAAAGCTTATCGATACCG |
| oAF799 | gtcgaggtcggcgaccag |
| oAF1069 | cgcctgacgccccatttagggtcagaaatcgtc |
| oAF1070 | cagttcgtcagctctcgtcgtcatcactcctcatc |
| oAF1071 | gatgaggagtgatgacgacgagagctgacgaactg |
| oAF1072 | CGggcggccgccctaggctgaggagctgagcc |
| oAF1073 | gattctagattgttcgtatgcgcgctg |
| oAF1074 | gacatcgattcagcttccggttccg |
| oAF1196 | cggtagcgggcgacctgcatcgtcatcactcctc |
| oAF1197 | atgcaggtcgcccgctaccgcac |
| oAF1198 | cttgaccttcttgggcatcgtcatcactcctc |
| oAF1199 | cgatgcccaagaaggtcaagccgc |
| oAF1291 | gccgccgGCtacgcactgggacatg |
| oAF1292 | ccagtgcgtaGCcggcggcttgaccttcttg |
