# Supplementary material for: Structure and function of the mycobacterial transcription initiation complex with the essential regulator RbpA
Source: eLife. 2017 Jan 9;6:e22520. doi: 10.7554/eLife.22520 (PMC5302886; doi:10.7554/eLife.22520)
Supplement: Supplementary file 10. — DOI: http://dx.doi.org/10.7554/eLife.22520.021 [file elife-22520-supp10.docx]

**Supplementary file 9. Plasmids used in generating *Msm* strains.**

| Plasmid name | relevant features | relevant details of construction |
| --- | --- | --- |
| pAJF441 | 3'rpoC-mCitrine-STII hyg | Harris et al., 2014 |
| pAJF527 | 3'rpoC-ppx-10his hyg oriE | in-fusion reaction 3'rpoC-ppx (oAF021/oAF796, pAJF441), ppx-10his-vector (oAF797/oAF020, pAJF441), and pAJF441 linearized with NdeI/HindIII |
| pAJF672 | attB::rbpA kan | rbpA (oAF1073/1074, MC2155 gDNA ) cut with XbaI/ClaI and ligated to pMV306kan cut with same |
| pAJF674 | rbpA KO flanks, hyg, galK, sacB, oriE | in-fusion reaction with KO front flank (oAF1069/1070, MC2155 gDNA), KO back flank (oAF1071/1072, MC2155 gDNA) and pAJF067 linearized with SpeI/NdeI |
| pAJF679 | attB:rbpA(28-114) kan | in-fusion reaction with 5'rbpA (306knfwd/oAF1196, pAJF672), 3'rbpA (oAF1197/oAF765, pAJF672), and pMV306kan linearized with XbaI/ClaI |
| pAJF680 | attB:rbpA(72-114) kan | in-fusion reaction with 5'rbpA (306knfwd/oAF1198, pAJF672), 3'rbpA (oAF1199/oAF765, pAJF672), and pMV306kan linearized with XbaI/ClaI |
| pAJF685 | attB::rbpA strep | rbpA cut from pAJF672 with XbaI/ClaI and ligated to pDB60 cut with same |
| pAJF736 | attB:rbpA(R79A) kan | in-fusion reaction of 5'rbpA (306knfwd/oAF1292, pAJF685), 3'rbpA (oAF1291/765, pAJF685) and pMV306kn linearized with XbaI/ClaI |
| pMV306kn | attB:kan | Lab Stock |
| pDB60 | attB:strep | Lab Stock |
| pAJF067 | galK sacB hyg oriE | Fay and Glickman, 2014 |
